# Oculopharyngeal muscular dystrophy or oculopharyngeal distal myopathy: case report^☆^

**DOI:** 10.1016/j.bjorl.2015.07.019

**Published:** 2015-11-05

**Authors:** Marilia Yuri Maeda, Tais Yuri Hashimoto, Isabella Christina Oliveira Neto, Luciano Rodrigues Neves

**Affiliations:** a Universidade Federal de São Paulo (UNIFESP), Fellowship em Otorrinolaringologia Pediátrica, São Paulo, SP, Brazil; b Universidade Federal de São Paulo (UNIFESP), Fellowship em Laringologia, São Paulo, SP, Brazil; c Universidade Federal de São Paulo (UNIFESP), Departamento de Fonoaudiologia, São Paulo, SP, Brazil; d Universidade Federal de São Paulo (UNIFESP), Departamento de ORL-CCP, São Paulo, SP, Brazil; e Universidade Nove de Julho (UNINOVE), São Paulo, SP, Brazil

## Introduction

Oculopharyngeal muscular dystrophy (OPMD) is a genetic disease with a predominantly autosomal dominant pattern, linked to the PABPN1 gene. OPMD progresses with a clinical picture of progressive ptosis, dysphagia, and weakness of the proximal muscles of the limbs.^1^

The disease usually begins in the fifth or sixth decade of life. In its recessive form, symptoms have a later onset are usually mild; in these cases the diagnosis becomes more difficult, and there may be confusion with symptoms of other diseases associated with aging.^2^

A Canadian study estimated the prevalence of OPMD at 1:1000.^3^ Most cases reported in the literature present a family history of neuromuscular disease, but there are rare cases where it was not possible to establish this association, and the appropriate explanation is the occurrence of mutation.^1^ The literature describes oculopharyngeal distal myopathy (OPDM) as a controversial entity: it is considered by some as a distinct disorder from OPMD, and by others as a variant of this disease.^4^

Thevathasan et al. report that early-onset ophthalmoparesis is a frequent finding in OPDM. The involvement of limb muscles initially occurs distally, and tibialis anterior muscle and intrinsic muscles of the hands are the most commonly affected. Lu et al. add that, in OPMD, the symptomatology begins most often in young adults, with a severe facial muscle weakness.

To carry out a proper investigation of OPMD and its differentiation from a variety of differential diagnoses (mitochondrial disorders, Nonaka myopathy, and OPDM, among others), it is critical to perform tests such as antibody anti-acetylcholine receptor dosage, serum lactate curve, electromyography, muscle biopsy, and genetic testing evaluation.^4^

The histopathological findings of both diseases (OPMD and OPDM) include nonspecific dystrophic myopathic abnormalities and the presence of vacuoles and tubular-filamentous intramuscular nuclear inclusions; the latter is larger in cases of OPDM (>8 nm).^5^

Genetic tests showed that in patients with OPDM, a repeating pattern of base pairs (“GCG”) in PABPN1 gene does not occur – a finding present in cases of OPMD.^4^

Among the muscle changes detected, dysphagia is the most concerning symptom, because it evidences a progressive weakening of the esophageal and pharyngeal muscles, which are responsible for the swallowing process. The degree of dysphagia presented by the patient is an important prognostic factor of the disease, as these patients progress to malnutrition.^6^

The curative treatment of OPMD is still unknown. However, medical or surgical treatments can be carried out in order to improve the patient's quality of life.

In the case of dysphagia, it is feasible to introduce changes in food consistency, as well as facilitating maneuvers and speech therapy for swallowing. When the established therapy no longer appears to be effective, or in face of a significant weight loss or recurrent aspiration pneumonia, there may be an indication for surgical treatment, e.g., cricopharyngeal myotomy or even gastrostomy.^6^

This study aimed to report a clinical case of a patient with significant muscle disorder and with a deltoid muscle biopsy compatible with OPMD. However, despite the diagnostic efforts, it was not possible to distinguish this disease from OPDM.

## Case report

ARS, 62-years old retired male, was assessed at this service because of complaints of long-time progressive dysphagia.

The patient reported the onset of progressive symmetrical ptosis at the age of 36 years. He was then evaluated and diagnosed with neuromuscular disease compatible with OPMD or OPDM (presence of rimmed vacuoles at the deltoid muscle biopsy material).

The patient was referred to the Department of Ophthalmology for surgery, aiming at a frontal suspension of his eyes, in order to facilitate the vision.

**Figure 1:**
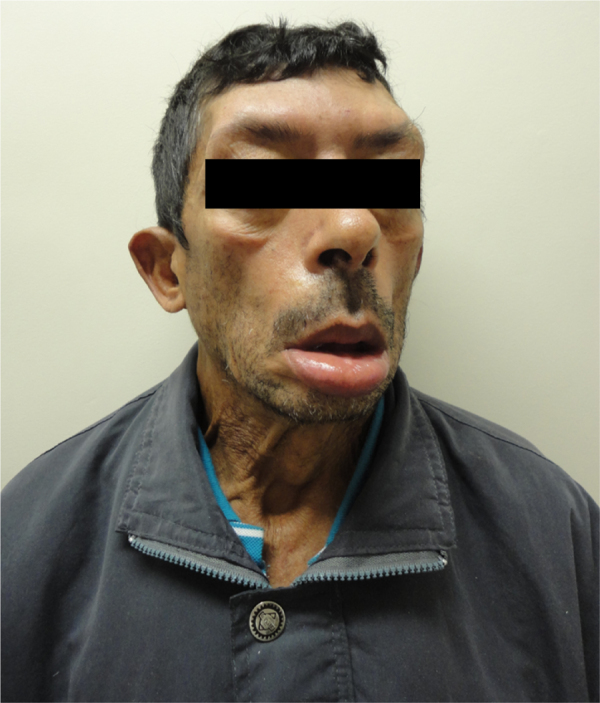
Patient's front view.

**Figure 2:**
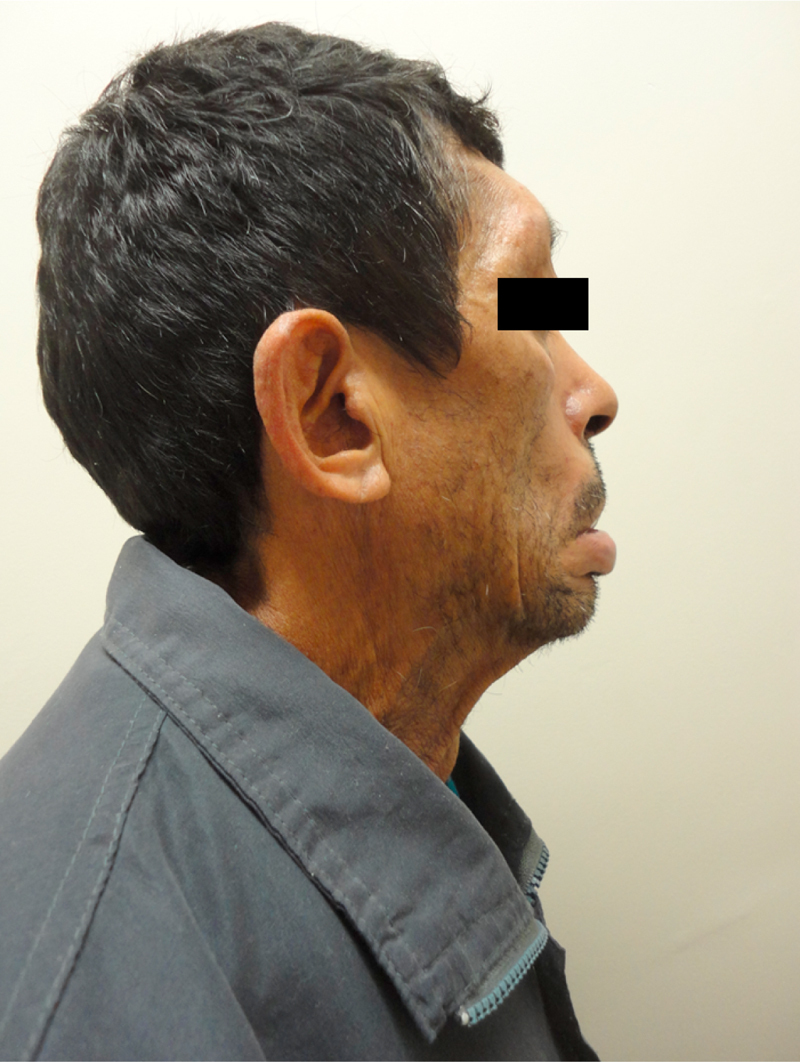
Patient's lateral view.

At 52 years of age, the patient reported the onset of dysphagia (choking on solid foods, need to clear his throat, and fluid intake after food intake). He also mentioned increasing difficulty in speaking and moving the muscles of the face and eyes, and reported the progression of symptoms (Figure 1, Figure 2).

**Figure 3:**
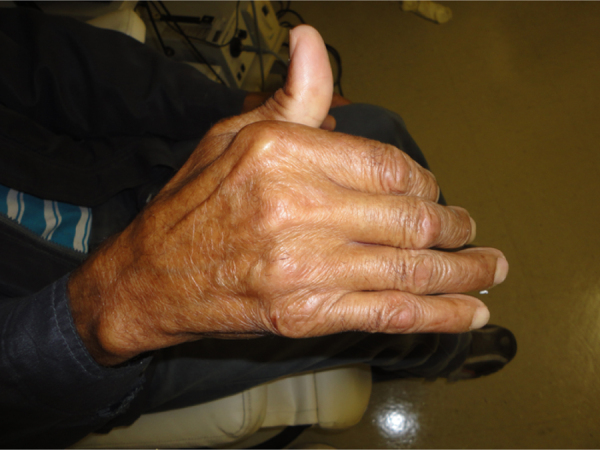
Claw hand.

At age 57 years, the patient had trouble raising his right arm and also opening his right hand, which was in a “claw” position, in addition to a general weakness of his legs that resulted in several falls (Fig. 3).

At that time, the patient said that his lips began to “fall” (*sic*), along with possible episodes of urinary incontinence.

Since the onset of symptoms, the patient reported a weight loss of around 13 kg and two episodes of pneumonia.

Family history revealed that the patient has seven siblings, two of whom have bilateral ptosis (one sister with onset of ptosis at age 69 years and one brother with onset at age 74 years). One of his four children has bilateral ptosis, which began at age 37 years.

The patient was assessed regarding his dysphagia complaints.

Swallowing endoscopy with the use of various food types and amounts (liquid, paste, and solid) detected a moderate oropharyngeal dysphagia with no visualization of penetration or food aspiration. Drooling caused by difficulty swallowing was also noted.

The patient was treated with speech therapy for deglutition, and botulinum toxin was applied in his salivary glands to reduce saliva production.

## Discussion

Despite controversies in the literature, van der Sluijs et al. reported in their article the main clinical features of OPMD^7^: a slowly progressive bilateral ptosis, weakness of proximal limbs, and progressive dysphagia.

These authors report that the abovementioned findings have a late onset; while in OPDM, the onset of symptoms occurs earlier (between 15 and 20 years of age), with an initial manifestation of tibialis anterior muscle weakness, or of bilateral ptosis.^7^

They also report that, in these patients, an intense hypotonia of the muscles of the face and of the extrinsic muscles of the eyes is noteworthy, which results in ptosis and limitation of ocular and facial movements, with a more severe and earlier occurrence than in cases of OPMD.^7^

Minami et al.^8^ discussed case reports of OPMD with involvement of distal muscles in the literature; these authors reported that it would be extremely difficult to distinguish OPMD from OPDM by simply analyzing the clinical features.

In that study, after an evaluation of PABPN1 gene, the authors obtained results regarding the presence of repetitions of the base pairs ‘GCG’ present in OPMD.^8^

In the clinical picture discussed in the present study, ptosis started at a young age (at 36 years of age). Furthermore, the patient showed muscle alterations in the face, extrinsic muscles of the eyes, right hand, and lower limbs.

Together with dysphagia, these signs and symptoms indicate a possible diagnosis of OPDM. However, the proximal muscles (scapular and pelvic girdles) were affected, which makes the diagnosis of OPMD possible.

**Figure 4:**
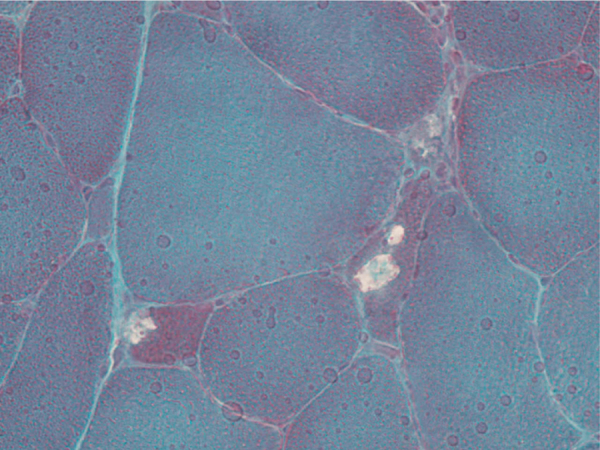
Rimmed vacuoles at the deltoid muscle biopsy (modified Gomori stain, 350×).

**Figure 5:**
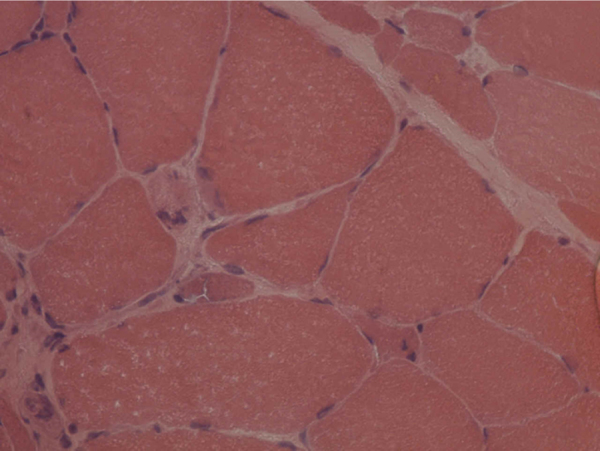
Presence of polygonal muscular atrophy and rimmed vacuoles (hematoxylin–eosin stain, 350×).

A deltoid muscle biopsy allowed detection of atrophic polygonal muscle fibers and of intrasarcoplasmic rimmed vacuoles. The presence of rimmed vacuoles is characteristic of both diseases; thus, it is not possible to differentiate between OPMD and OPDM (Figure 4, Figure 5).

Unfortunately, genetic testing to aid in diagnosis is not available at this service. As already foreseen by Minami et al.,^8^ it was not possible to differentiate these two diseases (OPMD and OPDM) based on the clinical information available.

Progressive dysphagia is seen in both diseases, and it may become severe and cause frequent aspiration pneumonia. Because this patient exhibited moderate oropharyngeal dysphagia with drooling, the authors enacted a clinical management protocol that included: speech therapy for deglutition; exercises to improve oral motor skills; change in food consistency; and chemical xerostomia with botulinum toxin application.

To date, there is no treatment for both diseases; thus, therapy should focus on the correction of any deficits presented, aiming at a better quality of life.

In addition to improving the dynamics of deglutition, the introduction of speech therapy for deglutition aimed to correct unwanted postures. Both the retroflexion of the head and frontal muscle contraction are known compensatory mechanisms for correction of ptosis, which has a negative effect on swallowing, worsening a pre-existing dysphagia. A slight bending of the head can improve the oropharyngeal phase of swallowing, reducing the meal time, the waste, and the risk of aspiration.^9^

The use of botulinum toxin in this patient's salivary glands aimed to decrease saliva production, resulting in less volume for swallowing. To date, this patient is still being followed-up for his dysphagia.

## Conclusion

In this study, the authors presented and described a case of a rare neuromuscular disease, with an important impact on the swallowing process.

## Conflicts of interest

The authors declare no conflicts of interest.
